# The Prognostic Value of Combined Smoking and Alcohol Consumption Habits for the Estimation of Cause-Specific Mortality in Middle-Age and Elderly Population: Results from a Long-Term Cohort Study in Lithuania

**DOI:** 10.1155/2017/9654314

**Published:** 2017-10-19

**Authors:** Dalia Luksiene, Abdonas Tamosiunas, Dalia Virviciute, Ricardas Radisauskas

**Affiliations:** ^1^Laboratory of Population Studies, Institute of Cardiology, Medical Academy, Lithuanian University of Health Sciences, Kaunas, Lithuania; ^2^Department of Environmental and Occupational Medicine, Public Health Faculty, Medical Academy, Lithuanian University of Health Sciences, Kaunas, Lithuania; ^3^Department of Preventive Medicine, Public Health Faculty, Medical Academy, Lithuanian University of Health Sciences, Kaunas, Lithuania

## Abstract

**Aim:**

To evaluate the prognostic value of combined smoking and alcohol consumption habits for the estimation of cause-specific mortality risk in middle-age and elderly population.

**Methods:**

The study presents data from the four surveys. A random sample of 6,729 subjects aged 35–64 years was selected for statistical analysis. During the follow-up of 31 years (1983–2014), there were 2,158 deaths from any cause. Multivariate Cox's proportional hazards models were used to estimate hazard ratios (HR) for all-cause mortality and Competing Risk Regression analysis was used to estimate subdistribution hazard risk (SHR) for cause-specific mortality.

**Results:**

Smoking clearly increased the risk of all-cause mortality and mortality from cancer and cardiovascular disease (CVD), but alcohol use had little effect in men aged 35–64 years. However, heavy alcohol consumption (>14 units/week) increased the risk of all-cause mortality and mortality from external causes in the never-smokers men group who drank alcohol of 1–14 units/week (HR 2 = 1.57 and SHR 2 = 2.40, resp.).

**Conclusions:**

The smoking habits and alcohol consumption are modifiable risk factors, and thus efforts to support abstinence from alcohol and smoking use should be a public health priority.

## 1. Introduction

Numerous studies have shown the relationships between smoking and an increased risk of mortality from cardiovascular diseases (CVD) and cancer [1, 2]. Alcohol has been identified as a leading risk factor for death and disability globally [3]. However, some meta-analyses have shown that regular light drinking appears to have little effect on overall mortality and may be protective against coronary heart disease (CHD) [4, 5]. Many studies analyzed these two risk factors separately [2, 6–8]. In reality, many people both smoke and drink. Few studies have examined the combined effects of smoking and drinking on mortality [9–11]. The results from a population-based cohort study of Chinese men have shown that light and moderate alcohol consumption reduced mortality from CVD, yet this beneficial effect was offset by cigarette smoking [9].

In Lithuania, the incidence and mortality rates of noncommunicable diseases, especially CVD, are higher than in most European countries, especially when comparing to high-income Western European countries [12]. Lithuania is characterized by having one of the most detrimental drinking patterns in the European Union [13]. Thus, alcohol consumption and cigarette smoking may be differentially associated with the risk of mortality in Lithuania. We hypothesized that alcohol consumption and cigarette smoking are strongly positively associated with the risk of mortality and have multiplicative interaction. The aim of this study was to evaluate the prognostic value of combined smoking and alcohol consumption habits for the estimation of cause-specific mortality risk in middle-age and elderly population.

## 2. Materials and Methods

The article presents data from four surveys. The first, the second, and the third clinical examinations within the framework of the Multinational Monitoring of Trends and Determinants in Cardiovascular Disease (MONICA) study [14] were performed in 1983-1984, 1986-1987, and 1992-1993. The fourth survey was conducted in 2001-2002 in accordance with the MONICA study protocol. These surveys were carried out in Kaunas city (Lithuania) with the population of 348,624. All four random samples of men and women aged 35–64 years, stratified by sex and age, were randomly selected from the Kaunas population register data. The response rates in the surveys varied between 58.6% and 70.2%. Data on 6,729 subjects examined for alcohol and smoking habits and analyzed for other risk factors were approved for statistical analysis. Data on 125 subjects were excluded from the statistical analysis due to incomplete information about the analyzed covariates. All respondents provided written informed consent.

A clinical examination was carried out in each survey. Blood pressure (BP), weight and height measurements, and laboratory analyses (total cholesterol) were conducted following the same methodology, and the same or comparable questionnaires were used. The body mass index (BMI) was calculated as the weight in kilograms divided by the height in meters squared (kg/m^2^).

In all surveys, CVD was determined according to the following criteria: CHD was determined by (1) a documented history of myocardial infarction (MI) and/or ischemic changes on an electrocardiogram (ECG) coded by the Minnesota codes (MC) 1-1 or 1-2 [15]; (2) angina pectoris defined by G. Rose's questionnaire (without MI and/or MC 1-1 or 1-2) [16]; (3) ECG findings (MC 1-3, 4-1, 4-2, 4-3, 5-1, 5-2, 5-3, 6-1, 6-2, 7-1, or 8-3 (without MI and/or MC 1-1 or 1-2, and without angina pectoris)). Stroke was determined using the question “Has a doctor ever told you that you have had a stroke?”

Diabetes mellitus was assessed by responses to the question “Have you ever been told by a doctor that you have diabetes? If, yes, how are you treated? Are you currently taking insulin and/or tablets?”

### 2.1. Covariates

Data on the respondents' age, education, marital status, smoking, and alcohol consumption habits were collected using a standard questionnaire. Education was classified into three education levels: incomplete secondary, secondary, and college or university. Marital status was classified into four categories: single, married, divorced, or widowed.

Smoking habits were assessed according to the current smoking status by responses to the question “Do you smoke cigarettes?” Subjects who answered “No, I have never smoked” were classified as nonsmokers, and subjects who answered “No, I smoked in the past, but I stopped” were classified as former smokers. Subjects who smoked at least one cigarette per day were classified as current smokers.

Alcohol consumption habits were assessed using a standard questionnaire. Respondents reported the quantity of spirits, beer, and wine usually consumed per week. According to the recommendations of the Handbook of Alcoholism [17], the responses were converted into units of alcohol, assuming the measure of the spirits consumed to be standard alcohol units (drinks): a bottle (0.5 L) of beer to be two units and a bottle of wine (0.7 L) to be six units. According to the European guidelines for CVD prevention [18] and 2015–2020 Dietary Guidelines for Americans [19], alcohol consumption was classified as none, 1–14 units per week, and more than 14 units/week in the men's group, and as none, 1–7 units/week, and more than 7 units/week in the women's group. Depending on their alcohol consumption and smoking habits, the participants were allocated to one of nine groups.

### 2.2. Follow-Up

The participants were followed up from the beginning of each clinical examination until January 1, 2015, and data on mortality were extracted from the regional mortality register. The causes of death were grouped as all-causes, CVD, cancer, and external causes. The first group consisted of deaths from all-causes: 001-E999: codes of the 9th revision of the International Classification of Diseases (ICD-9) (until January 1, 1997) and A00-Z99: codes of ICD-10 (after January 1, 1997). The second group consisted of deaths from CVD (390-458: codes of ICD-9 and I00-I99: codes of ICD-10). The third group consisted of deaths from cancer (140-239: codes of ICD-9 and C00-C96: codes of ICD-10), and the fourth group consisted of deaths from external causes (E800-E999: codes of ICD-9 and V01-Y98: codes of ICD-10). During 1983–2014, there were 2,158 deaths from any cause (1,292 men and 866 women), 820 deaths from CVD (excluding those with previous CVD at entry) (502 men and 318 women), 618 deaths from cancer (339 men and 279 women), and 146 deaths from external causes (115 men and 31 women). The mean duration of the follow-up was 21.21 ± 8.6 years.

### 2.3. Statistical Analysis

Descriptive characteristics (prevalence rates, means, and SD) were calculated for variables separately for men and women.

The association between smoking and alcohol consumption and the risk of all-cause mortality were investigated using Cox proportional hazards regression analysis, and hazard ratios (HR) were estimated. Also, the one minus survival function curves were estimated for all-cause mortality according to the combined modalities of alcohol and smoking consumption.

In survival analysis, traditional approaches such as Cox proportional hazards regression and Kaplan–Meier survival analysis are suitable to describe risk of all-cause deaths. However, these methods can overestimate risk of cause-specific death by failing to account for the competing risk of deaths from other causes. The recent scientific data show that the Kaplan–Meier survival analysis tended to result in an overestimate of the incidence of the outcome over time, while the Competing Risks Regression approach will tend to result in unbiased estimation of the incidence of the primary outcome over time [20]. In studies of elder individuals in which a substantial number of participants die during a long follow-up, the Cumulative Incidence Competing Risk estimate and Competing Risk Regression should be used to determine incidence and effect estimates [21–23]. Thus, in this study to evaluate influence of smoking and alcohol consumption on the cumulative incidence of cause-specific (CVD, cancer, and external causes of death) mortality Competing Risk Regression analysis was used, and subdistribution hazard ratios (SHR) were estimated. Also, the crude cumulative incidence curves were estimated for CVD, cancer mortality, and mortality from external causes according to the combined modalities of alcohol and smoking consumption.

Women were not included into this analysis due to small numbers of events and the number of women who were current smokers and drinkers. First, the association between smoking and alcohol consumption and changes in mortality between the surveys was evaluated using the likelihood ratio test, and the results were not statistically significant (*P* > 0.05). Thus, we calculated the association between smoking and alcohol consumption and the risk of mortality throughout the four surveys. HR represented changes in all-cause mortality and SHR represented changes in mortality from CVD, cancer, and external causes of death between different smoking and alcohol consumption groups. HR and SHR were adjusted by age (HR 1, SHR 1), and by age, total cholesterol level, BMI, systolic blood pressure (SBP), diabetes mellitus type 2, education level, and marital status (HR 2, SHR 2).

The reference groups were the following: never-smokers who reported drinking no alcohol were taken as reference group one (Table 4), and never-smokers who reported drinking 1–14 units of alcohol per week were taken as reference group two (Table 5).

Competing Risk analysis was carried out using STATA/MP version 13 software and all other analyses were carried out using SPSS version 13.

## 3. Results

During the 31-year follow-up period, 1,292 men (41.7%) and 866 (24.7%) women died. The predicted unadjusted risks (HR and SHR) for cause-specific mortality according to the main noncommunicable risk factors are shown in Tables 1 and 2. The age, smoking habits (former and current), heavy alcohol consumption, lower education level, marital status (widowed), diabetes mellitus type 2, increased total cholesterol, SBP, and BMI increased risk for all-cause mortality in men (Table 1) and in women (except for smoking and alcohol consumption habits) (Table 2). The age, smoking habits (former and current), lower education level, marital status (widowed), diabetes mellitus type 2, increased total cholesterol, SBP, and BMI increased the cumulative incidence of death from CVD in men (Table 1) and in women (Table 2). The age, smoking habits (former and current), and lower education level increased the cumulative incidence of death from cancer in men (Table 1) and in women (except for smoking habits and education level) (Table 2). The heavy alcohol consumption and marital status (divorced) increased the cumulative incidence of death from external causes in men (Table 1). In addition, in women group none alcohol consumption increased risk for all-cause mortality and mortality from CVD and cancer compared to women who drank moderate amounts of alcohol (1–14 units/week) (Table 2).

The numbers and proportions of men and women in each alcohol use and smoking group are shown in Table 3. Only 6.3% of men were smokers and consumed more than 14 units of alcohol per week during the baseline survey. About one-third (30.2%) of men were smokers and drank 1–14 units/week. The survey showed a similar proportion (33.7%) of men who were never-smokers and drank moderate amounts of alcohol (1–14 units/week). Most of women (72.5%) were never-smokers and drank moderate amounts of alcohol (1–7 units/week); only 0.30% of women were smokers and drank more than 7 units of alcohol per week during the baseline survey. Thus, because the number of women who were current or former smokers and drinkers was limited, further data analysis of the risk of mortality in relation to smoking and alcohol consumption was performed only in men.

**Figure 1:**
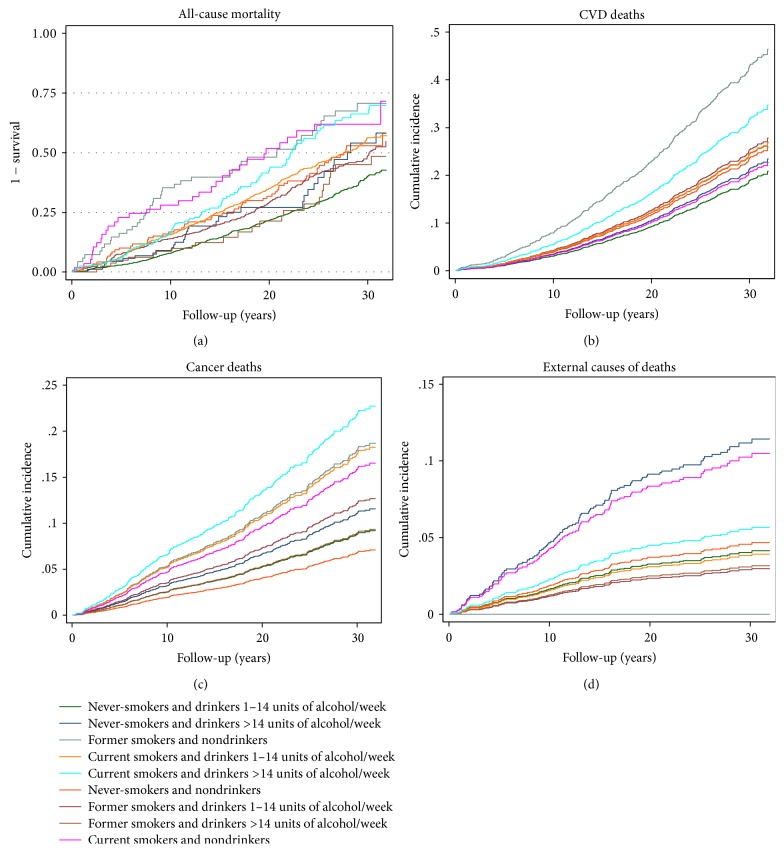
The one minus survival function curves for all-cause mortality (a), and the crude cumulative incidence curves for CVD mortality (b), cancer mortality (c), and mortality from external causes (d) according to the combined modalities of alcohol and smoking consumption.

Figure 1 presents the one minus survival function curves for all-cause mortality (a) and the crude cumulative incidence curves were estimated for CVD (b), cancer mortality (c), and mortality from external causes (d) according to the combined modalities of alcohol and smoking consumption. During the 31-year follow-up period, the probability of all-causes mortality was the highest in current smokers and nondrinkers men group (near 70%) and the lowest in never-smokers men group who reported drinking 1–14 units of alcohol per week (near 40%). The probability of mortality from CVD was the highest in former smokers and nondrinkers men group (near 47%) and the lowest in never-smokers men group who reported drinking 1–14 units of alcohol per week (near 20%). The probability of mortality from cancer was the highest in men group who smoked and drank more than 14 units of alcohol per week (near 23%) and the lowest in never-smokers and nondrinkers men group (near 6%). The probability of mortality from external causes was the highest in men group who never smoked and drank more than 14 units of alcohol per week (near 12%) and the lowest in former smokers and nondrinkers men group (0%).

Men who smoked and drank more than 14 units of alcohol per week had the highest age-adjusted risk of all-cause mortality (HR 1 = 2.33), compared to never-smokers and nondrinkers (Table 4). The cumulative incidence of death from cancer and CVD was higher in men group who smoked and drank more than 14 units of alcohol per week compared to never-smokers and nondrinkers (SHR 1 = 4.88 and SHR 1 = 1.96, resp.). After adjustment by age, total cholesterol level, BMI, SBP, diabetes mellitus type 2, education level, and marital status, men who smoked and drank more than 14 units of alcohol per week had the highest risk of all-cause mortality risk (HR 2 = 2.13) and the cumulative incidence of death from cancer (SHR 2 = 4.17) was higher in this men group compared to never-smokers and nondrinkers. Adjustment for these risk factors attenuated the risk, but the pattern remained. Former smokers and nondrinkers also had a higher age-adjusted risk (HR 1) and additional adjusted risk (HR 2) of all-cause mortality and the cumulative incidence of death from cancer (SHR 1 and SHR 2, resp.), compared to never-smokers and nondrinkers (Table 4). These results show that for all-cause mortality and mortality from cancer, there was a clear effect of smoking, but alcohol consumption had little effect.

Similar results were obtained when the combined effect of smoking and drinking on mortality was analyzed with a different reference group—never-smokers who reported drinking 1–14 units of alcohol per week (Table 5). Men who both smoked and drank more than 14 units of alcohol per week had the highest adjusted risk of all-cause mortality, compared to never-smokers who reported drinking 1–14 units of alcohol per week (HR 2 = 2.55). Also, the cumulative incidence of death from cancer and CVD was higher in men group who smoked and drank more than 14 units of alcohol per week, compared to never-smokers who reported drinking 1–14 units of alcohol per week (SHR 2 = 2.56 and SHR 2 = 2.02, resp.). However, heavy alcohol consumption (more than 14 units/week) increased the age-adjusted risk of all-cause mortality and mortality from external causes, compared to the respective risk observed in the group of never-smokers who reported drinking 1–14 units of alcohol per week (HR 1 = 1.71 and SHR 1 = 2.86, resp.). After additional adjustment, this significant impact on mortality remained (HR 2 = 1.57 and SHR 2 = 2.40, resp.).

## 4. Discussion

Two of the biggest threats to the world population's health come from the negative effects of tobacco and alcohol use [24]. Over the last few decades, it has become clear that heavy use of tobacco and alcohol leads to serious health consequences and is related to an increased risk of mortality. In our large longitudinal study, we analyzed the combined impact of alcohol and smoking habits on the risk of cause-specific mortality (from any cause, CVD, cancer, or external causes) in urban population aged 35–64 years.

Alcohol and cigarette smoking each have an individual effect on the risk of mortality, but, when combined, they act synergistically [24]. Epidemiological studies have demonstrated that, in adults, high rates of smoking strongly correlate with alcohol use [25, 26]. A case-control study compared the natural history of cigarette smoking in alcoholic and nonalcoholic populations, and the results of this study showed that 83% of alcoholics were smokers compared to 34% of the nonalcoholic subjects [27]. Data from the Public Health Survey in Stockholm showed that total consumption of alcohol as well as binge drinking at least monthly was associated with the use of all kinds of tobacco among both men and women [28]. Our data indicated that during screening, among 3,230 men aged 35–64, only 6.3% were smokers and drank more than 14 units of alcohol per week, and 30.2% were smokers and drank 1–14 units/week. Meanwhile, in Scotland, in men aged 35–64 years, the proportion of men who smoked and drank more than 14 units/week was 21.0% [11], which is by about 3 times higher than in our population of men. However, in Scotland, 8.5% of the studied men were never-smokers and nondrinkers [11], while in our population, the proportion of such men was only 3.4%. There is a lack of data on women's smoking and drinking habits. The majority of this kind of studies are carried out only in men. Our data indicated that most women (72.5%) were never-smokers and drank moderate amounts of alcohol (1-7 units/week), and only about 4.9% of the women were smokers and drank 1–7 units/week. However, this number was insufficient to perform the Cox proportional hazards regression and Competing Risk Regression analysis, and therefore women were not included in this analysis.

A longitudinal 30-year-long study conducted in Scotland also analyzed the combined effects of smoking and alcohol consumption on the cause-specific risk of mortality [11]. The participants were 5,771 men aged 35–64 years, and the results showed that smoking and drinking 15 and more units of alcohol per week were the riskiest behavior for all-causes of death in the male population of Scotland [11].

A study of 64,515 Chinese men aged from 30 to 89 years who were screened between 1996 and 2000, with an average of 4.6 years of follow-up, related smoking, use of alcohol, and mortality [9]. The highest all-cause mortality was seen in heavy drinkers and heavy smokers. There was a protective effect of moderate drinking for all-cause and CVD mortality, which was stronger in nonsmokers than ever-smokers. Heavy drinking was associated with an increased mortality from cancer, and there was no protective effect of moderate drinking on cancer mortality even in nonsmokers [9].

The results of our study showed that alcohol use and smoking both contributed to the risk of mortality in men. Our study indicated that men who smoked and drank more than 14 units of alcohol per week had the highest risk of all-cause mortality and mortality from CVD and cancer, compared to never-smokers who did not drink alcohol. Nevertheless, male former smokers and nondrinkers also had a higher risk of all-cause mortality and mortality from CVD and cancer, compared to never-smokers and nondrinkers. Thus, these results showed that, in the population of Lithuanian men, smoking had stronger effects on the risk of all-cause mortality and mortality from CVD and cancer than alcohol consumption did. Smoking had stronger effects than alcohol did on most of the investigated causes. Current smokers had a consistently higher risk of mortality. Former smokers had a lower risk of mortality from all-causes, CVD, and cancer than current smokers did, showing the beneficial effects of smoking cessation, but they had a higher risk of mortality, compared to never-smokers. Data from a meta-analysis study corroborates and expands evidence from previous studies in showing that smoking is a strong independent risk factor of CVD mortality even at older age: random-effects meta-analysis of the association of the smoking status with CVD mortality yielded a summary HR of 2.07 (95% CI 1.82–2.36) for current smokers and 1.37 (95% CI 1.25–1.49) for former smokers, compared to never-smokers [29]. Thus, the results from our study present that the cumulative incidence of death from CVD was higher in men group who smoked and drank more than 14 units of alcohol per week, compared to never-smokers who reported drinking 1–14 units of alcohol per week (SHR 2 = 2.02).

Similar results were obtained when we analyzed the combined effects of smoking and drinking on mortality, but the reference group was never-smokers who reported drinking 1–14 units of alcohol per week. In addition, heavy alcohol consumption (more than 14 units per week) increased the risk of all-cause mortality and mortality from external causes, compared to the respective risk in male never-smokers who reported drinking 1–14 units of alcohol per week. The analysis of the structure of deaths associated with heavy alcohol use and their contribution to overall mortality in Northwest Slovakia from 2005 to 2012 showed that external causes dominated among cases of death associated with heavy alcohol use [30].

Although there is enough literature establishing an association between alcohol consumption and tobacco use, the mechanisms explaining this relationship are not completely understood. Some investigators explained that the interaction between alcohol consumption and cigarette smoking can be explained by genetic factors, neurobiological mechanisms, conditioning mechanisms, and psychosocial factors [31]. The study findings in Chinese adult male population showed that smoking habits were associated not only with alcohol consumption, but also with unhealthy lifestyle habits: heavy smokers consumed significantly less vegetables, fruit, milk, and other dairy products, spent significantly more time watching television, and slept and exercised less, compared to never-smokers or former smokers [32].

### 4.1. Strengths

The strengths of our study are its long follow-up period and the ability to adjust for several risk factors and analyze the impact of smoking and alcohol use on several causes of death. In the presented analyses, we have adjusted for a range of potential confounding variables, including age, sex, total cholesterol, BMI, SBP, diabetes mellitus type 2, education level, and marital status. In addition, our study includes the prospective character, which makes selection and information bias unlikely. Also, in this study for the evaluation of influence of smoking and alcohol consumption on the cumulative incidence of cause-specific (CVD, cancer, and external causes of death) mortality Competing Risk Regression analysis was used.

### 4.2. Limitations

Several limitations of this study should be considered when interpreting the results. First, these samples of Kaunas city population are not necessarily representative of the whole adult Lithuanian population. Second, the number of women who were current or former smokers and were drinking alcohol was limited, and for this reason, the analysis of data on the risk of mortality by smoking and alcohol consumption categories was performed only in men. Third, alcohol consumption and smoking habits were self-reported and may thus have been underestimated. As alcohol and smoking habits were reported by participants at baseline surveys, we do not know if these lifestyle habits were continued or changed during the follow-up. If some former smokers took up smoking again, this would increase the risk of mortality in former smokers. Alternatively, some subjects who were current smokers at baseline survey might have given up smoking later, which would have decreased the risk of mortality in the current smokers group. Our main analyses did not take into account the amount of cigarettes smoked in the current and former smoker's groups. Although adjustments were made for several covariates, the study did not record other possible covariates such as dietary intake, family history of disease, or adequate information on physical activity.

## 5. Conclusions

The study showed that, in the Lithuanian male population aged 35–64 years, smoking clearly increased the risk of all-cause mortality and mortality from CVD and cancer, but alcohol use had little effect. However, in the never-smokers group, heavy alcohol consumption (>14 units/week) increased the risk of all-cause mortality and mortality from external causes, compared to the respective risk in men who were nonsmokers and reported drinking 1–14 units of alcohol per week. Thus, smoking and alcohol consumption are associated with cause-specific mortality risk significantly and independently of other well-known sociodemographics and biological risk factors. Alcohol consumption and smoking habits are modifiable risk factors, and thus efforts to support abstinence from smoking and alcohol use should be a public health priority in Lithuania.

## Figures and Tables

**Table 1:** Hazard ratios (HR) and subdistribution hazard ratios for cause-specific mortality according to the risk factors in men group (univariate analyses).

Characteristics	All-cause deaths			CVD deaths^†^			Cancer			External causes of death		
	Events	HR (95% CI)	*P*	Events	SHR (95% CI)	*P*	Events	SHR (95% CI)	*P*	Events	SHR (95% CI)	*P*
Age, years	1292	1.08 (1.07–1.09)	<0.0001	502	1.08 (1.06–1.09)	<0.0001	339	1.07 (1.05–1.08)	<0.0001	115	0.99 (0.97–1.01)	0.459
Smoking habits												
Never	430	1		173	1		92	1		52	1	
Former	303	1.38 (1.19–1.60)	<0.0001	122	1.42 (1.13–1.78)	0.003	72	1.45 (1.06–1.97)	0.019	17	0.58 (0.33–0.99)	0.049
Current	559	1.65 (1.45–1.87)	<0.0001	207	1.36 (1.11–1.66)	0.003	175	2.18 (1.70–2.81)	<0.0001	46	0.97 (0.65–1.44)	0.868
Alcohol consumption category												
None	133	1		38	1		24	1		10	1	
1–14 units/week	1007	0.63 (0.52–0.75)	<0.0001	403	0.80 (0.53–1.12)	0.193	271	1.08 (0.71–1.65)	0.722	86	0.81 (0.42–1.57)	0.533
>14 units/week	152	0.84 (0.67–1.06)	0.149	61	1.03 (0.68–1.54)	0.896	44	1.44 (0.87–2.38)	0.159	19	1.41 (0.65–3.05)	0.385
Alcohol consumption category												
None	133	1.59 (1.33–1.91)	<0.0001	38	1.25 (0.89–1.74)	0.193	24	0.93 (0.61–1.42)	0.722	10	1.23 (0.64–2.39)	0.533
1–14 units/week	1007	1		403	1		271	1		86	1	
>14 units/week	152	1.34 (1.13–1.59)	0.001	61	1.29 (0.98–1.68)	0.070	44	1.33 (0.97–1.83)	0.077	19	1.74 (1.06–2.85)	0.029
Total cholesterol, mmol/L	1292	1.05 (1.0–1.10)	0.05	502	1.13 (1.05–1.21)	0.001	339	0.96 (0.87–1.07)	0.474	115	0.98 (0.80–1.20)	0.838
SBP, mmHg	1292	1.02 (1.01–1.02)	<0.0001	502	1.02 (1.02–1.03)	<0.0001	339	1.00 (0.99–1.01)	0.132	115	0.99 (0.99–1.01)	0.632
BMI, kg/m^2^	1292	1.02 (1.01–1.03)	0.007	502	1.06 (1.04–1.09)	<0.0001	339	0.95 (0.92–0.99)	0.005	115	0.89 (0.84–0.94)	<0.0001
Diabetes mellitus type 2												
No	1248	1		488	1		333	1		110	1	
Yes	44	2.67 (1.98–3.61)	<0.0001	14	2.12 (1.22–3.67)	0.007	6	0.89 (0.39–2.03)	0.787	5	2.23 (0.92–5.40)	0.076
Education level												
Incomplete secondary	585	1		231	1		166	1		46	1	
Secondary	458	0.54 (0.48–0.61)	<0.0001	163	0.52 (0.42–0.63)	<0.0001	113	0.52 (0.41–0.66)	<0.0001	50	0.83 (0.55–1.23)	0.352
University or college	249	0.41 (0.35–0.47)	<0.0001	108	0.48 (0.38–0.61)	<0.0001	60	0.40 (0.30–0.54)	<0.0001	19	0.47 (0.28–0.80)	0.005
Marital status												
Married	1175	1		455	1		318	1		99	1	
Single	33	1.02 (0.72–1.44)	0.923	13	1.03 (0.57–1.79)	0.929	9	1.00 (0.51–1.94)	0.989	2	0.69 (0.17–2.80)	0.607
Widowed	31	2.48 (1.74–3.55)	<0.0001	14	2.69 (1.54–4.71)	<0.0001	5	0.98 (0.40–2.39)	0.963	4	2.41 (0.88–6.55)	0.086
Divorced	53	1.25 (0.95–1.65)	0.108	20	1.17 (0.74–1.86)	0.494	7	0.56 (0.26–1.19)	0.133	10	2.74 (1.44–5.24)	0.002

CI: confidence interval, CVD: cardiovascular diseases, BMI: body mass index, SBP: systolic blood pressure, and SD: standard deviation. *CVD deaths*^†^ excluded those with previous CVD at the entry. SHR: subdistribution hazard risk; follow-up, years, mean (SD) 20.1 (8.75).

**Table 2:** Hazard ratios (HR) and subdistribution hazard ratios for cause-specific mortality according to the risk factors in women group (univariate analyses).

Characteristics	All-cause deaths			CVD deaths^†^			Cancer			External causes of death		
	Events	HR (95% CI)	*P*	Events	SHR (95% CI)	*P*	Events	SHR (95% CI)	*P*	Events	SHR (95% CI)	*P*
Age, years	866	1.10 (1.09–1.12)	<0.0001	318	1.14 (1.13–1.16)	<0.0001	279	1.04 (1.03–1.05)	<0.0001	31	1.07 (1.02–1.12)	0.007
Smoking habits												
Never	801	1		292	1		262	1		27	1	
Former	25	1.18 (0.79–1.75)	0.427	13	1.72 (1.00–2.97)	0.049	5	0.66 (0.27–1.61)	0.364	1	1.22 (0.17–8.92)	0.847
Current	40	1.19 (0.87–1.63)	0.288	13	0.98 (0.57–1.71)	0.956	12	1.01 (0.57–1.81)	0.969	3	2.28 (0.69–7.51)	0.177
Alcohol consumption category												
None	259	1		85	1		75	1		8	1	
1–14 units/week	603	0.60 (0.52–0.69)	<0.0001	233	0.66 (0.52–0.85)	0.001	201	0.73 (0.56–0.95)	0.019	23	0.77 (0.35–1.71)	0.517
>14 units/week	4	0.38 (0.14–1.02)	0.056	0	—	—	3	0.98 (0.31–3.12)	0.978	0	—	—
Alcohol consumption category												
None	259	1.67 (1.45–1.93)	<0.0001	85	1.51 (1.17–1.93)	0.001	75	1.38 (1.05–1.80)	0.019	8	1.30 (0.59–2.89)	0.517
1–14 units/week	603	1		233	1		201	1		23	1	
>14 units/week	4	0.64 (0.24–1.70)	0.369	0	—	—	3	1.35 (0.43–4.21)	0.602	0	—	—
Total cholesterol, mmol/L	866	1.13 (1.08–1.19)	<0.0001	318	1.18 (1.10–1.27)	<0.001	279	1.07 (0.99–1.17)	0.091	31	0.87 (0.66–1.16)	0.354
SBP, mmHg	866	1.02 (1.01–1.02)	<0.0001	318	1.02 (1.02–1.03)	<0.0001	279	1.00 (1.00–1.01)	0.033	31	1.00 (0.99–1.02)	0.438
BMI, kg/m^2^	866	1.05 (1.04–1.07)	<0.0001	318	1.07 (1.05–1.08)	<0.0001	279	1.03 (1.01–1.04)	0.002	31	1.03 (0.98–1.09)	0.196
Diabetes mellitus type 2												
No	808	1		294	1		269	1		30	1	
Yes	58	3.98 (3.05–5.20)	<0.0001	24	4.31 (2.76–6.74)	<0.0001	10	1.35 (0.71–2.58)	0.355	1	1.17 (0.16–8.67)	0.875
Education level												
Incomplete secondary	448	1		179	1		109	1		21	1	
Secondary	310	0.54 (0.46–0.62)	<0.0001	109	0.44 (0.35–0.56)	<0.0001	111	0.85 (0.65–1.10)	0.220	7	0.22 (0.09–0.54)	0.001
University or college	108	0.32 (0.26–0.40)	<0.0001	30	0.22 (0.15–0.32)	<0.0001	59	0.81 (0.59–1.11)	0.143	3	0.20 (0.06–0.66)	0.009
Marital status												
Married	671	1		241	1		240	1		22	1	
Single	32	0.74 (0.52–1.05)	0.090	13	0.86 (0.49–1.51)	0.588	7	0.45 (0.21–0.96)	0.038	1	0.71 (0.10–5.31)	0.741
Widowed	97	1.65 (1.34–2.05)	<0.0001	38	2.10 (1.49–2.96)	<0.0001	15	0.63 (0.37–1.07)	0.088	3	1.34 (0.40–4.48)	0.633
Divorced	66	1.08 (0.83–1.39)	0.577	26	1.17 (0.78–1.76)	0.449	17	0.75 (0.46–1.25)	0.272	5	2.59 (0.99–6.81)	0.053

CI: confidence interval, CVD: cardiovascular diseases, BMI: body mass index, SBP: systolic blood pressure, and SD: standard deviation. *CVD deaths*^†^ excluded those with previous CVD at the entry. SHR: subdistribution hazard risk; follow-up, years, mean (SD) 22.35 (7.79).

**Table 3:** Number and proportion^†^ of men and women by smoking and alcohol consumption category.

Drinking status	Smoking status		
	Never	Former	Current
Men (*n* = 3230)			
None	119 (3.40)	68 (1.90)	57 (1.70)
1–14 units/week	1088 (33.7)	587 (17.4)	945 (30.2)
>14 units/week	88 (2.80)	81 (2.60)	197 (6.30)
Women (*n* = 3499)			
None	646 (17.8)	15 (0.40)	10 (0.30)
1–7 units/week	2523 (72.5)	92 (2.60)	163 (4.90)
>7 units/week	31 (0.90)	9 (0.30)	1 (0.30)

^†^Adjusted by age.

**Table 4:** Risk of mortality during the 31-year follow-up by smoking and alcohol consumption category in men (the reference groups: never-smokers who reported drinking no alcohol).

Drinking status	Smoking status		
	Never	Former	Current
*All-cause deaths*			
None			
Number of deaths	58	43	32
HR 1 (95% CI)	1	**1.63 (1.10–2.42)**	**2.15 (1.40–3.13)**
HR 2 (95% CI)	1	**1.60 (1.07–2.38)**	**1.99 (1.29–3.08)**
1–14 units/week			
Number of deaths	340	236	431
HR 1 (95% CI)	0.80 (0.61–1.06)	0.99 (0.74–1.32)	**1.59 (1.21–2.10)**
HR 2 (95% CI)	0.84 (0.63–1.11)	0.98 (0.73–1.31)	**1.66 (1.25–2.21)**
>14 units/week			
Number of deaths	32	24	96
HR 1 (95% CI)	1.37 (0.89–2.12)	0.94 (0.58–1.52)	**2.33 (1.67–3.24)**
HR 2 (95% CI)	1.31 (0.85–2.03)	0.88 (0.54–1.42)	**2.13 (1.52–2.99)**
*CVD deaths* ^†^			
None			
Number of deaths	18	13	7
SHR 1 (95% CI)	1	2.00 (0.97–4.15)	0.96 (0.39–2.35)
SHR 2 (95% CI)	1	1.62 (0.74–3.57)	0.91 (0.38–2.18)
1–14 units/week			
Number of deaths	144	97	162
SHR 1 (95% CI)	0.91 (0.55–1.50)	1.11 (0.67–1.85)	1.39 (0.84–2.29)
SHR 2 (95% CI)	0.85 (0.51–1.42)	0.95 (0.56–1.60)	1.41 (0.84–2.36)
>14 units/week			
Number of deaths	11	12	38
SHR 1 (95% CI)	1.17 (0.55–2.53)	1.18 (0.58–2.39)	**1.96 (1.10–3.49)**
SHR 2 (95% CI)	1.01 (0.47–2.17)	1.01 (0.49–2.08)	1.72 (0.95–3.14)
*Cancer deaths*			
None			
Number of deaths	7	10	7
SHR 1 (95% CI)	1	**2.68 (1.00–7.18)**	**3.04 (1.04–8.88)**
SHR 2 (95% CI)	1	**2.72 (1.01–7.33)**	2.73 (0.93–7.98)
1–14 units/week			
Number of deaths	78	57	136
SHR 1 (95% CI)	1.62 (0.75–3.50)	1.99 (0.91–4.34)	**3.84 (1.80–8.20)**
SHR 2 (95% CI)	1.63 (0.76–3.49)	1.93 (0.88–4.22)	**3.49 (1.64–7.47)**
>14 units/week			
Number of deaths	7	5	32
SHR 1 (95% CI)	2.32 (0.81–6.61)	1.70 (0.53–5.43)	**4.88 (2.16–11.0)**
SHR 2 (95% CI)	2.26 (0.79–6.44)	1.57 (0.49–5.03)	**4.17 (1.83–9.53)**
*External causes of death*			
None			
Number of deaths	5	0	5
SHR 1 (95% CI)	1	—	2.28 (0.65–8.02)
SHR 2 (95% CI)	1	—	1.52 (0.42–5.51)
1–14 units/week			
Number of deaths	39	15	32
SHR 1 (95% CI)	0.87 (0.34–2.22)	0.63 (0.23–1.73)	0.82 (0.31–2.11)
SHR 2 (95% CI)	1.02 (0.39–2.67)	0.69 (0.25–1.99)	0.68 (0.26–1.78)
>14 units/week			
Number of deaths	8	2	9
SHR 1 (95% CI)	2.48 (0.80–7.65)	0.66 (0.13–3.41)	1.19 (0.40–3.57)
SHR 2 (95% CI)	2.45 (0.78–7.69)	0.68 (0.13–3.35)	0.86 (0.29–2.56)

*CVD deaths* ^†^ mortality from CVD excluded those with previous CVD at the entry. CI: confidence interval, CVD: cardiovascular diseases, HR: hazard ratio, and SHR: subdistribution hazard risk. *HR 1, SHR 1* adjusted for age. *HR 2, SHR 2* adjusted for age, total cholesterol, body mass index, systolic blood pressure, type 2 diabetes mellitus, education level, and marital status.

**Table 5:** Risk of mortality during the 31-year follow-up by smoking and alcohol consumption category in men (the reference groups: never-smokers who reported drinking 1–14 units of alcohol per week).

Drinking status	Smoking status		
	Never	Former	Current
*All-cause deaths*			
None			
Number of deaths	58	43	32
HR 1 (95% CI)	1.25 (0.94–1.65)	**2.03 (1.48–2.79)**	**2.68 (1.86–3.85)**
HR 2 (95% CI)	1.19 (0.90–1.58)	**1.91 (1.38–2.62)**	**2.38 (1.65–3.43)**
1–14 units/week			
Number of deaths	340	236	431
HR 1 (95% CI)	1	**1.23 (1.04–1.46)**	**1.98 (1.72–2.29)**
HR 2 (95% CI)	1	1.17 (0.99–1.38)	**1.99 (1.71–2.30)**
>14 units/week			
Number of deaths	32	24	96
HR 1 (95% CI)	**1.71 (1.19–2.46)**	1.17 (0.78–1.78)	**2.90 (2.31–3.65)**
HR 2 (95% CI)	**1.57 (1.09–2.26)**	1.05 (0.69–1.59)	**2.55 (2.01–3.22)**
*CVD deaths* ^†^			
None			
Number of deaths	18	13	7
SHR 1 (95% CI)	1.10 (0.67–1.81)	**2.20 (1.23–3.92)**	1.05 (0.48–2.30)
SHR 2 (95% CI)	1.17 (0.70–1.95)	**1.90 (1.00–3.63)**	1.06 (0.50–2.24)
1–14 units/week			
Number of deaths	144	97	162
SHR 1 (95% CI)	1	1.22 (0.95–1.57)	**1.52 (1.22–1.91)**
SHR 2 (95% CI)	1	1.11 (0.86–1.43)	**1.65 (1.31–2.08)**
>14 units/week			
Number of deaths	11	12	38
SHR 1 (95% CI)	1.29 (0.69–2.41)	1.29 (0.75–2.24)	**2.15 (1.49–3.09)**
SHR 2 (95% CI)	1.19 (0.65–2.17)	1.18 (0.67–2.06)	**2.02 (1.38–2.97)**
*Cancer deaths*			
None			
Number of deaths	7	10	7
SHR 1 (95% CI)	0.62 (0.29–1.33)	1.65 (0.83–3.30)	1.88 (0.84–4.21)
SHR 2 (95% CI)	0.61 (0.29–1.32)	1.67 (0.83–3.37)	1.67 (0.74–3.77)
1–14 units/week			
Number of deaths	78	57	136
SHR 1 (95% CI)	1	1.23 (0.87–1.73)	**2.37 (1.79–3.14)**
SHR 2 (95% CI)	1	1.19 (0.84–1.68)	**2.15 (1.62–2.84)**
>14 units/week			
Number of deaths	7	5	32
SHR 1 (95% CI)	1.43 (0.66–3.11)	1.05 (0.42–2.64)	**3.01 (2.00–4.53)**
SHR 2 (95% CI)	1.39 (0.63–3.03)	0.96 (0.38–2.43)	**2.56 (1.67–3.93)**
*External causes of death*			
None			
Number of deaths	5	0	5
SHR 1 (95% CI)	1.15 (0.45–2.95)	—	**2.62 (1.02–6.77)**
SHR 2 (95% CI)	0.98 (0.37–2.57)	—	1.49 (0.55–4.07)
1–14 units/week			
Number of deaths	39	15	32
SHR 1 (95% CI)	1	0.72 (0.40–1.32)	0.94 (0.59–1.50)
SHR 2 (95% CI)	1	0.69 (0.37–1.26)	0.67 (0.41–1.09)
>14 units/week			
Number of deaths	8	2	9
SHR 1 (95% CI)	**2.86 (1.34–6.10)**	0.76 (0.18–3.13)	1.37 (0.67–2.82)
SHR 2 (95% CI)	**2.40 (1.12–5.18)**	0.67 (0.17–2.56)	0.84 (0.41–1.74)

*CVD deaths* ^†^ mortality from CVD excluded those with previous CVD at the entry. CI: confidence interval, CVD: cardiovascular diseases, HR: hazard ratio, and SHR: subdistribution hazard risk. *HR 1, SHR 1* adjusted for age. *HR 2, SHR 2* adjusted for age, total cholesterol, body mass index, systolic blood pressure, type 2 diabetes mellitus, education level, and marital status.
